# Supplementary material for: Pyruvate Dehydrogenase Kinase 1 inhibition mediated oxidative phosphorylation enhancement in cartilage promotes osteoarthritis progression
Source: BMC Musculoskelet Disord. 2023 Jul 20;24:597. doi: 10.1186/s12891-023-06585-6 (PMC10357736; doi:10.1186/s12891-023-06585-6)
Supplement: Supplementary file 1 — Additional file 1. [file 12891_2023_6585_MOESM1_ESM.zip › KEGG.Gsea.1653622432464/KEGG_NOTCH_SIGNALING_PATHWAY.html]

Details for gene set KEGG\_NOTCH\_SIGNALING\_PATHWAY[GSEA]

|  || Dataset | 55457\_collapsed\_to\_symbols.cls.cls #OA\_versus\_normal.cls.cls #OA\_versus\_normal\_repos |
| Phenotype | cls.cls#OA\_versus\_normal\_repos |
| Upregulated in class | normal |
| GeneSet | KEGG\_NOTCH\_SIGNALING\_PATHWAY |
| Enrichment Score (ES) | -0.34696594 |
| Normalized Enrichment Score (NES) | -1.2637849 |
| Nominal p-value | 0.15151516 |
| FDR q-value | 0.30507568 |
| FWER p-Value | 1.0 |
Table: GSEA Results Summary

  

Fig 1: Enrichment plot: KEGG\_NOTCH\_SIGNALING\_PATHWAY      
 Profile of the Running ES Score & Positions of GeneSet Members on the Rank Ordered List

  

| SYMBOL | TITLE | RANK IN GENE LIST | RANK METRIC SCORE | RUNNING ES | CORE ENRICHMENT || 1 | PSEN1 | presenilin 1 [Source:HGNC Symbol;Acc:HGNC:9508] | 425 | 0.695 | 0.0276 | No |
| 2 | NCSTN | nicastrin [Source:HGNC Symbol;Acc:HGNC:17091] | 666 | 0.601 | 0.0614 | No |
| 3 | PSENEN | "presenilin enhancer, gamma-secretase subunit [Source:HGNC Symbol;Acc:HGNC:30100]" | 1529 | 0.424 | 0.0313 | No |
| 4 | RFNG | RFNG O-fucosylpeptide 3-beta-N-acetylglucosaminyltransferase [Source:HGNC Symbol;Acc:HGNC:9974] | 2646 | 0.286 | -0.0306 | No |
| 5 | DVL2 | dishevelled segment polarity protein 2 [Source:HGNC Symbol;Acc:HGNC:3086] | 2693 | 0.280 | -0.0098 | No |
| 6 | MFNG | MFNG O-fucosylpeptide 3-beta-N-acetylglucosaminyltransferase [Source:HGNC Symbol;Acc:HGNC:7038] | 3442 | 0.207 | -0.0498 | No |
| 7 | APH1A | "aph-1 homolog A, gamma-secretase subunit [Source:HGNC Symbol;Acc:HGNC:29509]" | 4256 | 0.132 | -0.1015 | No |
| 8 | PSEN2 | presenilin 2 [Source:HGNC Symbol;Acc:HGNC:9509] | 4544 | 0.109 | -0.1143 | No |
| 9 | DVL1 | dishevelled segment polarity protein 1 [Source:HGNC Symbol;Acc:HGNC:3084] | 4612 | 0.104 | -0.1105 | No |
| 10 | KAT2B | lysine acetyltransferase 2B [Source:HGNC Symbol;Acc:HGNC:8638] | 4867 | 0.082 | -0.1230 | No |
| 11 | CTBP2 | C-terminal binding protein 2 [Source:HGNC Symbol;Acc:HGNC:2495] | 5066 | 0.067 | -0.1325 | No |
| 12 | SNW1 | SNW domain containing 1 [Source:HGNC Symbol;Acc:HGNC:16696] | 5472 | 0.035 | -0.1610 | No |
| 13 | LFNG | LFNG O-fucosylpeptide 3-beta-N-acetylglucosaminyltransferase [Source:HGNC Symbol;Acc:HGNC:6560] | 6161 | -0.017 | -0.2130 | No |
| 14 | NCOR2 | nuclear receptor corepressor 2 [Source:HGNC Symbol;Acc:HGNC:7673] | 6497 | -0.042 | -0.2354 | No |
| 15 | DTX2 | deltex E3 ubiquitin ligase 2 [Source:HGNC Symbol;Acc:HGNC:15973] | 6992 | -0.079 | -0.2669 | No |
| 16 | NUMB | NUMB endocytic adaptor protein [Source:HGNC Symbol;Acc:HGNC:8060] | 7180 | -0.095 | -0.2731 | No |
| 17 | MAML1 | mastermind like transcriptional coactivator 1 [Source:HGNC Symbol;Acc:HGNC:13632] | 7275 | -0.101 | -0.2717 | No |
| 18 | DTX4 | deltex E3 ubiquitin ligase 4 [Source:HGNC Symbol;Acc:HGNC:29151] | 7640 | -0.130 | -0.2886 | No |
| 19 | DLL3 | delta like canonical Notch ligand 3 [Source:HGNC Symbol;Acc:HGNC:2909] | 7702 | -0.134 | -0.2817 | No |
| 20 | NOTCH3 | notch receptor 3 [Source:HGNC Symbol;Acc:HGNC:7883] | 7894 | -0.147 | -0.2837 | No |
| 21 | HDAC1 | histone deacetylase 1 [Source:HGNC Symbol;Acc:HGNC:4852] | 7911 | -0.148 | -0.2721 | No |
| 22 | DVL3 | dishevelled segment polarity protein 3 [Source:HGNC Symbol;Acc:HGNC:3087] | 8756 | -0.213 | -0.3191 | No |
| 23 | RBPJ | recombination signal binding protein for immunoglobulin kappa J region [Source:HGNC Symbol;Acc:HGNC:5724] | 8800 | -0.217 | -0.3036 | No |
| 24 | HDAC2 | histone deacetylase 2 [Source:HGNC Symbol;Acc:HGNC:4853] | 9359 | -0.260 | -0.3243 | Yes |
| 25 | CIR1 | "corepressor interacting with RBPJ, CIR1 [Source:HGNC Symbol;Acc:HGNC:24217]" | 9640 | -0.287 | -0.3210 | Yes |
| 26 | KAT2A | lysine acetyltransferase 2A [Source:HGNC Symbol;Acc:HGNC:4201] | 9799 | -0.301 | -0.3071 | Yes |
| 27 | PTCRA | pre T cell antigen receptor alpha [Source:HGNC Symbol;Acc:HGNC:21290] | 9865 | -0.308 | -0.2853 | Yes |
| 28 | DTX3 | deltex E3 ubiquitin ligase 3 [Source:HGNC Symbol;Acc:HGNC:24457] | 10102 | -0.331 | -0.2747 | Yes |
| 29 | CTBP1 | C-terminal binding protein 1 [Source:HGNC Symbol;Acc:HGNC:2494] | 10188 | -0.339 | -0.2518 | Yes |
| 30 | RBPJL | recombination signal binding protein for immunoglobulin kappa J region like [Source:HGNC Symbol;Acc:HGNC:13761] | 10650 | -0.388 | -0.2538 | Yes |
| 31 | NOTCH2 | notch receptor 2 [Source:HGNC Symbol;Acc:HGNC:7882] | 10720 | -0.395 | -0.2247 | Yes |
| 32 | CREBBP | CREB binding protein [Source:HGNC Symbol;Acc:HGNC:2348] | 10881 | -0.414 | -0.2010 | Yes |
| 33 | JAG1 | jagged canonical Notch ligand 1 [Source:HGNC Symbol;Acc:HGNC:6188] | 10977 | -0.427 | -0.1712 | Yes |
| 34 | JAG2 | jagged canonical Notch ligand 2 [Source:HGNC Symbol;Acc:HGNC:6189] | 11039 | -0.435 | -0.1379 | Yes |
| 35 | NOTCH4 | notch receptor 4 [Source:HGNC Symbol;Acc:HGNC:7884] | 11441 | -0.495 | -0.1260 | Yes |
| 36 | EP300 | E1A binding protein p300 [Source:HGNC Symbol;Acc:HGNC:3373] | 11741 | -0.540 | -0.1022 | Yes |
| 37 | ADAM17 | ADAM metallopeptidase domain 17 [Source:HGNC Symbol;Acc:HGNC:195] | 11931 | -0.579 | -0.0664 | Yes |
| 38 | NOTCH1 | notch receptor 1 [Source:HGNC Symbol;Acc:HGNC:7881] | 12555 | -0.776 | -0.0472 | Yes |
| 39 | HES1 | hes family bHLH transcription factor 1 [Source:HGNC Symbol;Acc:HGNC:5192] | 12676 | -0.849 | 0.0174 | Yes |
Table: GSEA details [plain text format]

  

Fig 2: KEGG\_NOTCH\_SIGNALING\_PATHWAY      
 Blue-Pink O' Gram in the Space of the Analyzed GeneSet

  

Fig 3: KEGG\_NOTCH\_SIGNALING\_PATHWAY: Random ES distribution      
 Gene set null distribution of ES for **KEGG\_NOTCH\_SIGNALING\_PATHWAY**
